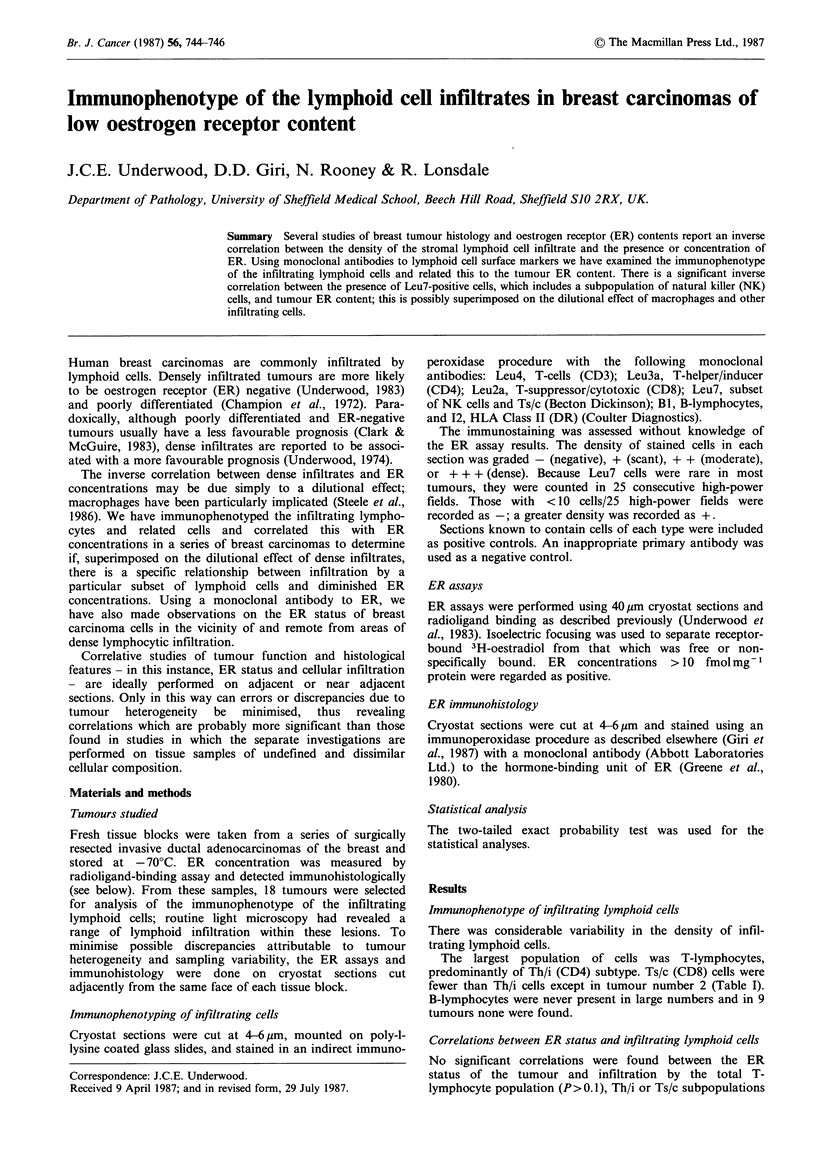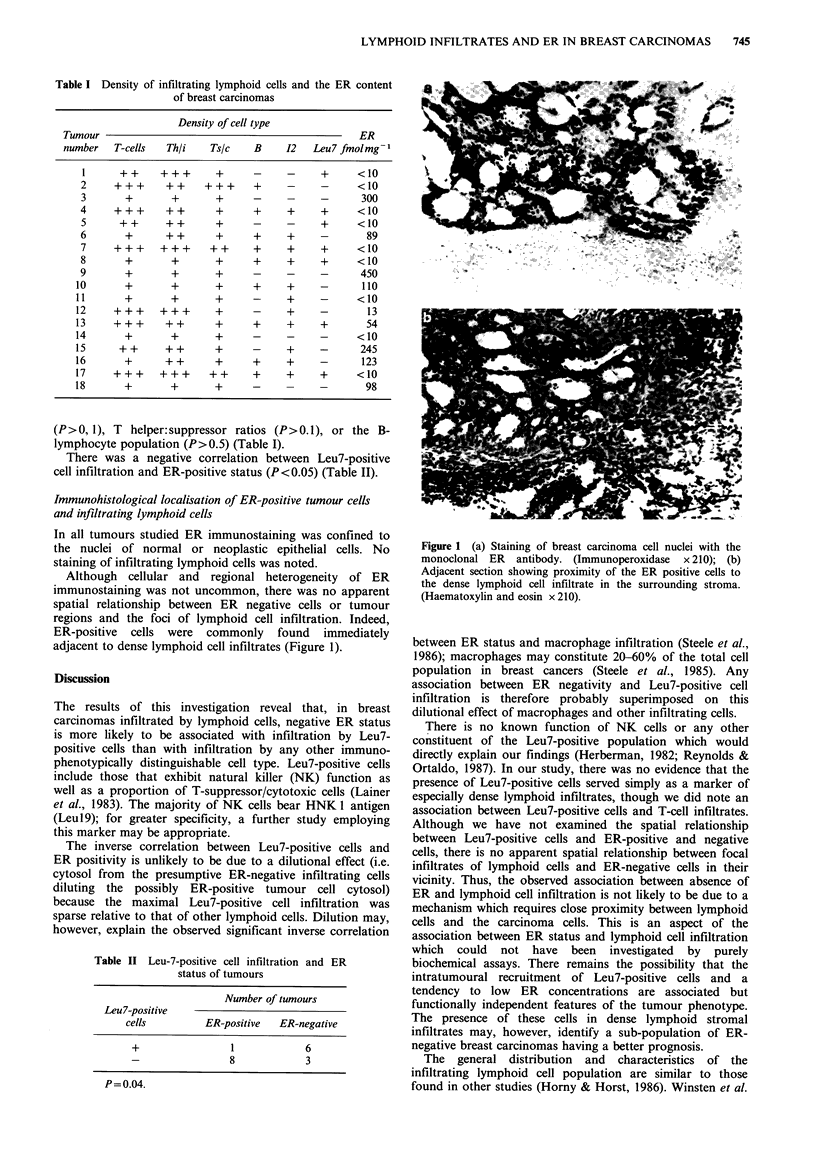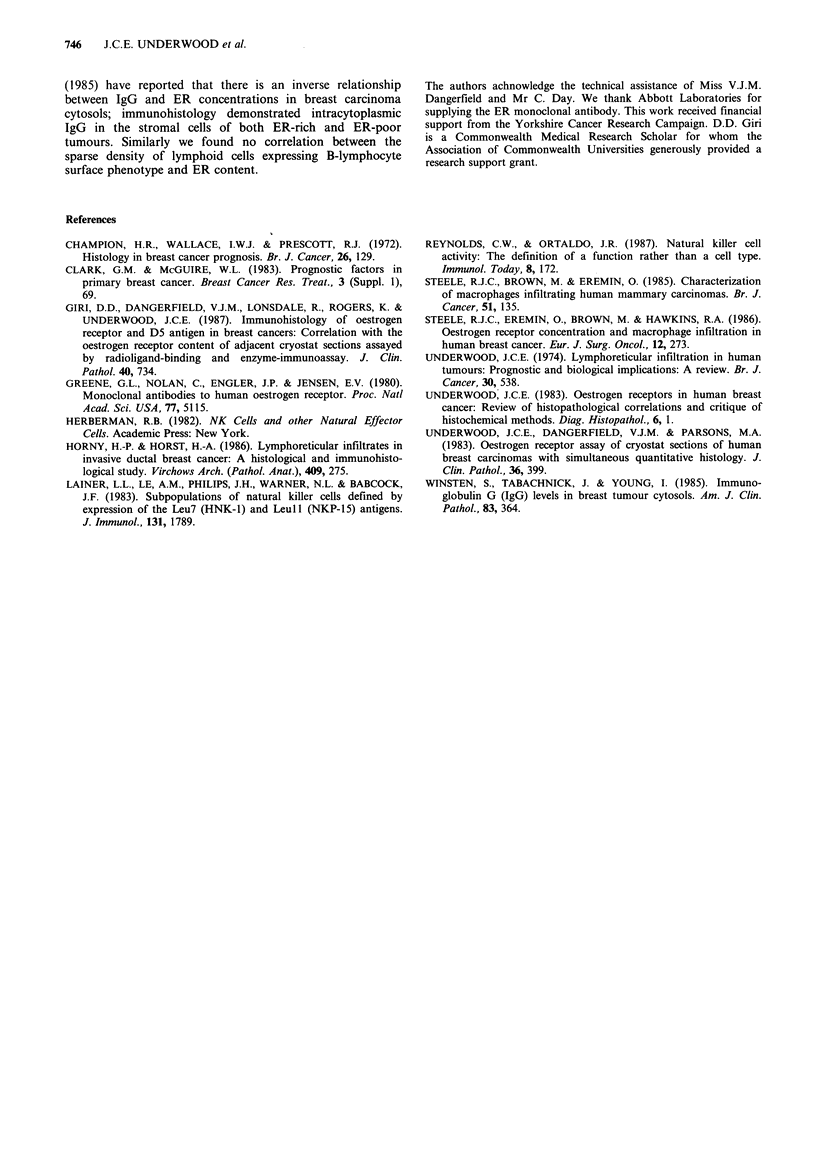# Immunophenotype of the lymphoid cell infiltrates in breast carcinomas of low oestrogen receptor content.

**DOI:** 10.1038/bjc.1987.281

**Published:** 1987-12

**Authors:** J. C. Underwood, D. D. Giri, N. Rooney, R. Lonsdale

**Affiliations:** Department of Pathology, University of Sheffield Medical School, UK.

## Abstract

**Images:**


*Br. J. Cancer* (1987) **56**, 744–746 © The Macmillan Press Ltd., 1987

### Immunophenotype of the lymphoid cell infiltrates in breast carcinomas of low oestrogen receptor content

J.C.E. Underwood, D.D. Giri, N. Rooney & R. Lonsdale

*Department of Pathology, University of Sheffield Medical School, Beech Hill Road, Sheffield S10 2RX, UK.*

**Summary** Several studies of breast tumour histology and oestrogen receptor (ER) contents report an inverse correlation between the density of the stromal lymphoid cell infiltrate and the presence or concentration of ER. Using monoclonal antibodies to lymphoid cell surface markers we have examined the immunophenotype of the infiltrating lymphoid cells and related this to the tumour ER content. There is a significant inverse correlation between the presence of Leu7-positive cells, which includes a subpopulation of natural killer (NK) cells, and tumour ER content; this is possibly superimposed on the dilutional effect of macrophages and other infiltrating cells.

Human breast carcinomas are commonly infiltrated by lymphoid cells. Densely infiltrated tumours are more likely to be oestrogen receptor (ER) negative (Underwood, 1983) and poorly differentiated (Champion *et al.*, 1972). Paradoxically, although poorly differentiated and ER-negative tumours usually have a less favourable prognosis (Clark & McGuire, 1983), dense infiltrates are reported to be associated with a more favourable prognosis (Underwood, 1974).

The inverse correlation between dense infiltrates and ER concentrations may be due simply to a dilutional effect; macrophages have been particularly implicated (Steele *et al.*, 1986). We have immunophenotyped the infiltrating lymphocytes and related cells and correlated this with ER concentrations in a series of breast carcinomas to determine if, superimposed on the dilutional effect of dense infiltrates, there is a specific relationship between infiltration by a particular subset of lymphoid cells and diminished ER concentrations. Using a monoclonal antibody to ER, we have also made observations on the ER status of breast carcinoma cells in the vicinity of and remote from areas of dense lymphocytic infiltration.

Correlative studies of tumour function and histological features – in this instance, ER status and cellular infiltration – are ideally performed on adjacent or near adjacent sections. Only in this way can errors or discrepancies due to tumour heterogeneity be minimised, thus revealing correlations which are probably more significant than those found in studies in which the separate investigations are performed on tissue samples of undefined and dissimilar cellular composition.

### Materials and methods

### *Tumours studied*

Fresh tissue blocks were taken from a series of surgically resected invasive ductal adenocarcinomas of the breast and stored at −70°C. ER concentration was measured by radioligand-binding assay and detected immunohistologically (see below). From these samples, 18 tumours were selected for analysis of the immunophenotype of the infiltrating lymphoid cells; routine light microscopy had revealed a range of lymphoid infiltration within these lesions. To minimise possible discrepancies attributable to tumour heterogeneity and sampling variability, the ER assays and immunohistology were done on cryostat sections cut adjacently from the same face of each tissue block.

### *Immunophenotyping of infiltrating cells*

Cryostat sections were cut at 4–6 $\mu$m, mounted on poly-l-lysine coated glass slides, and stained in an indirect immunoperoxidase procedure with the following monoclonal antibodies: Leu4, T-cells (CD3); Leu3a, T-helper/inducer (CD4); Leu2a, T-suppressor/cytotoxic (CD8); Leu7, subset of NK cells and Ts/c (Becton Dickinson); B1, B-lymphocytes, and I2, HLA Class II (DR) (Coulter Diagnostics).

The immunostaining was assessed without knowledge of the ER assay results. The density of stained cells in each section was graded − (negative), + (scant), + + (moderate), or + + + (dense). Because Leu7 cells were rare in most tumours, they were counted in 25 consecutive high-power fields. Those with <10 cells/25 high-power fields were recorded as −; a greater density was recorded as +.

Sections known to contain cells of each type were included as positive controls. An inappropriate primary antibody was used as a negative control.

### *ER assays*

ER assays were performed using 40 $\mu$m cryostat sections and radioligand binding as described previously (Underwood *et al.*, 1983). Isoelectric focusing was used to separate receptor-bound $^3$H-oestradiol from that which was free or non-specifically bound. ER concentrations >10 fmol mg$^{-1}$ protein were regarded as positive.

### *ER immunohistology*

Cryostat sections were cut at 4–6 $\mu$m and stained using an immunoperoxidase procedure as described elsewhere (Giri *et al.*, 1987) with a monoclonal antibody (Abbott Laboratories Ltd.) to the hormone-binding unit of ER (Greene *et al.*, 1980).

### *Statistical analysis*

The two-tailed exact probability test was used for the statistical analyses.

### Results

### *Immunophenotype of infiltrating lymphoid cells*

There was considerable variability in the density of infiltrating lymphoid cells.

The largest population of cells was T-lymphocytes, predominantly of Th/i (CD4) subtype. Ts/c (CD8) cells were fewer than Th/i cells except in tumour number 2 (Table I). B-lymphocytes were never present in large numbers and in 9 tumours none were found.

### *Correlations between ER status and infiltrating lymphoid cells*

No significant correlations were found between the ER status of the tumour and infiltration by the total T-lymphocyte population ($P$>0.1), Th/i or Ts/c subpopulations

Correspondence: J.C.E. Underwood.
Received 9 April 1987; and in revised form, 29 July 1987.

**Table I** Density of infiltrating lymphoid cells and the ER content of breast carcinomas

| Tumour number | *Density of cell type* T-cells | *Th/i* | *Ts/c* | *B* | *I2* | *Leu7* | *ER fmol mg*$^{-1}$ |
|---|---|---|---|---|---|---|---|
| 1 | ++ | +++ | + | − | − | + | <10 |
| 2 | +++ | ++ | +++ | + | − | − | <10 |
| 3 | + | + | + | − | − | − | 300 |
| 4 | +++ | ++ | + | + | + | + | <10 |
| 5 | ++ | ++ | + | − | − | + | <10 |
| 6 | + | ++ | + | + | + | − | 89 |
| 7 | +++ | +++ | ++ | + | + | + | <10 |
| 8 | + | + | + | + | + | + | <10 |
| 9 | + | + | + | − | − | − | 450 |
| 10 | + | + | + | + | + | − | 110 |
| 11 | + | + | + | − | + | − | <10 |
| 12 | +++ | +++ | + | − | + | − | 13 |
| 13 | +++ | ++ | + | + | + | + | 54 |
| 14 | + | + | + | − | − | − | <10 |
| 15 | ++ | ++ | + | − | + | − | 245 |
| 16 | + | ++ | + | + | + | − | 123 |
| 17 | +++ | +++ | ++ | + | + | + | <10 |
| 18 | + | + | + | − | − | − | 98 |

($P$>0, 1), T helper:suppressor ratios ($P$>0.1), or the B-lymphocyte population ($P$>0.5) (Table I).

There was a negative correlation between Leu7-positive cell infiltration and ER-positive status ($P$<0.05) (Table II).

*Immunohistological localisation of ER-positive tumour cells and infiltrating lymphoid cells*

In all tumours studied ER immunostaining was confined to the nuclei of normal or neoplastic epithelial cells. No staining of infiltrating lymphoid cells was noted.

Although cellular and regional heterogeneity of ER immunostaining was not uncommon, there was no apparent spatial relationship between ER negative cells or tumour regions and the foci of lymphoid cell infiltration. Indeed, ER-positive cells were commonly found immediately adjacent to dense lymphoid cell infiltrates (Figure 1).

### Discussion

The results of this investigation reveal that, in breast carcinomas infiltrated by lymphoid cells, negative ER status is more likely to be associated with infiltration by Leu7-positive cells than with infiltration by any other immunophenotypically distinguishable cell type. Leu7-positive cells include those that exhibit natural killer (NK) function as well as a proportion of T-suppressor/cytotoxic cells (Lainer *et al.*, 1983). The majority of NK cells bear HNK 1 antigen (Leu19); for greater specificity, a further study employing this marker may be appropriate.

The inverse correlation between Leu7-positive cells and ER positivity is unlikely to be due to a dilutional effect (i.e. cytosol from the presumptive ER-negative infiltrating cells diluting the possibly ER-positive tumour cell cytosol) because the maximal Leu7-positive cell infiltration was sparse relative to that of other lymphoid cells. Dilution may, however, explain the observed significant inverse correlation

**Table II** Leu-7-positive cell infiltration and ER status of tumours

| *Leu7-positive cells* | *Number of tumours* ER-positive | *ER-negative* |
|---|---|---|
| + | 1 | 6 |
| − | 8 | 3 |

$P$=0.04.

**Figure 1** (a) Staining of breast carcinoma cell nuclei with the monoclonal ER antibody. (Immunoperoxidase ×210); (b) Adjacent section showing proximity of the ER positive cells to the dense lymphoid cell infiltrate in the surrounding stroma. (Haematoxylin and eosin ×210).

between ER status and macrophage infiltration (Steele *et al.*, 1986); macrophages may constitute 20–60% of the total cell population in breast cancers (Steele *et al.*, 1985). Any association between ER negativity and Leu7-positive cell infiltration is therefore probably superimposed on this dilutional effect of macrophages and other infiltrating cells.

There is no known function of NK cells or any other constituent of the Leu7-positive population which would directly explain our findings (Herberman, 1982; Reynolds & Ortaldo, 1987). In our study, there was no evidence that the presence of Leu7-positive cells served simply as a marker of especially dense lymphoid infiltrates, though we did note an association between Leu7-positive cells and T-cell infiltrates. Although we have not examined the spatial relationship between Leu7-positive cells and ER-positive and negative cells, there is no apparent spatial relationship between focal infiltrates of lymphoid cells and ER-negative cells in their vicinity. Thus, the observed association between absence of ER and lymphoid cell infiltration is not likely to be due to a mechanism which requires close proximity between lymphoid cells and the carcinoma cells. This is an aspect of the association between ER status and lymphoid cell infiltration which could not have been investigated by purely biochemical assays. There remains the possibility that the intratumoural recruitment of Leu7-positive cells and a tendency to low ER concentrations are associated but functionally independent features of the tumour phenotype. The presence of these cells in dense lymphoid stromal infiltrates may, however, identify a sub-population of ER-negative breast carcinomas having a better prognosis.

The general distribution and characteristics of the infiltrating lymphoid cell population are similar to those found in other studies (Horny & Horst, 1986). Winsten *et al.*

(1985) have reported that there is an inverse relationship between IgG and ER concentrations in breast carcinoma cytosols; immunohistology demonstrated intracytoplasmic IgG in the stromal cells of both ER-rich and ER-poor tumours. Similarly we found no correlation between the sparse density of lymphoid cells expressing B-lymphocyte surface phenotype and ER content.

The authors achnowledge the technical assistance of Miss V.J.M. Dangerfield and Mr C. Day. We thank Abbott Laboratories for supplying the ER monoclonal antibody. This work received financial support from the Yorkshire Cancer Research Campaign. D.D. Giri is a Commonwealth Medical Research Scholar for whom the Association of Commonwealth Universities generously provided a research support grant.

### References

CHAMPION, H.R., WALLACE, I.W.J. & PRESCOTT, R.J. (1972). Histology in breast cancer prognosis. *Br. J. Cancer*, **26**, 129.

CLARK, G.M. & MCGUIRE, W.L. (1983). Prognostic factors in primary breast cancer. *Breast Cancer Res. Treat.*, **3** (Suppl. 1), 69.

GIRI, D.D., DANGERFIELD, V.J.M., LONSDALE, R., ROGERS, K. & UNDERWOOD, J.C.E. (1987). Immunohistology of oestrogen receptor and D5 antigen in breast cancers: Correlation with the oestrogen receptor content of adjacent cryostat sections assayed by radioligand-binding and enzyme-immunoassay. *J. Clin. Pathol.* **40**, 734.

GREENE, G.L., NOLAN, C., ENGLER, J.P. & JENSEN, E.V. (1980). Monoclonal antibodies to human oestrogen receptor. *Proc. Natl Acad. Sci. USA*, **77**, 5115.

HERBERMAN, R.B. (1982). *NK Cells and other Natural Effector Cells*. Academic Press: New York.

HORNY, H.-P. & HORST, H.-A. (1986). Lymphoreticular infiltrates in invasive ductal breast cancer: A histological and immunohistological study. *Virchows Arch.* (*Pathol. Anat.*), **409**, 275.

LAINER, L.L., LE, A.M., PHILIPS, J.H., WARNER, N.L. & BABCOCK, J.F. (1983). Subpopulations of natural killer cells defined by expression of the Leu7 (HNK-1) and Leu11 (NKP-15) antigens. *J. Immunol.*, **131**, 1789.

REYNOLDS, C.W., & ORTALDO, J.R. (1987). Natural killer cell activity: The definition of a function rather than a cell type. *Immunol. Today*, **8**, 172.

STEELE, R.J.C., BROWN, M. & EREMIN, O. (1985). Characterization of macrophages infiltrating human mammary carcinomas. *Br. J. Cancer*, **51**, 135.

STEELE, R.J.C., EREMIN, O., BROWN, M. & HAWKINS, R.A. (1986). Oestrogen receptor concentration and macrophage infiltration in human breast cancer. *Eur. J. Surg. Oncol.*, **12**, 273.

UNDERWOOD, J.C.E. (1974). Lymphoreticular infiltration in human tumours: Prognostic and biological implications: A review. *Br. J. Cancer*, **30**, 538.

UNDERWOOD, J.C.E. (1983). Oestrogen receptors in human breast cancer: Review of histopathological correlations and critique of histochemical methods. *Diag. Histopathol.*, **6**, 1.

UNDERWOOD, J.C.E., DANGERFIELD, V.J.M. & PARSONS, M.A. (1983). Oestrogen receptor assay of cryostat sections of human breast carcinomas with simultaneous quantitative histology. *J. Clin. Pathol.*, **36**, 399.

WINSTEN, S., TABACHNICK, J. & YOUNG, I. (1985). Immunoglobulin G (IgG) levels in breast tumour cytosols. *Am. J. Clin. Pathol.*, **83**, 364.